# Screening for Neurocognitive Deficits in Pediatrics—the Clinical Utility of the Pediatric Perceived Cognitive Functioning item bank

**DOI:** 10.1093/arclin/acaf065

**Published:** 2025-07-22

**Authors:** Marieke de Vries, Jan Pieter Marchal, Heleen Maurice-Stam, Berdien Mulder, Martha Grootenhuis, Femke K Aarsen, Andre B Rietman, Michiel A J Luijten, Kim J Oostrom

**Affiliations:** Department of Development & Education of Youth in Diverse Societies, Utrecht University, Heidelberglaan 1, 3584 CS, Utrecht, the Netherlands; School of Psychology, University of Nottingham Malaysia, Jalan Broga, Semenyih, Malaysia; Department of Child and Adolescent Psychiatry & Psychosocial Care, Emma Children's Hospital, Academic Medical Center, University of Amsterdam, Meibergdreef 9, 1105 AZ, Amsterdam, The Netherlands; Princess Maxima Center for Pediatric Oncology, Utrecht University, Heidelberglaan 25, 3584 CS Utrecht, the Netherlands; Department of Child and Adolescent Psychiatry & Psychosocial Care, Emma Children's Hospital, Academic Medical Center, University of Amsterdam, Meibergdreef 9, 1105 AZ, Amsterdam, The Netherlands; Princess Maxima Center for Pediatric Oncology, Utrecht University, Heidelberglaan 25, 3584 CS Utrecht, the Netherlands; Princess Maxima Center for Pediatric Oncology, Utrecht University, Heidelberglaan 25, 3584 CS Utrecht, the Netherlands; Department of Child and Adolescent Psychiatry/Psychology, Erasmus MC Sophia Children’s Hospital, Dr. Molewaterplein 40, 3015 GD Rotterdam, the Netherlands; Department of Child and Adolescent Psychiatry & Psychosocial Care, Emma Children's Hospital, Academic Medical Center, University of Amsterdam, Meibergdreef 9, 1105 AZ, Amsterdam, The Netherlands; Department of Child and Adolescent Psychiatry & Psychosocial Care, Emma Children's Hospital, Academic Medical Center, University of Amsterdam, Meibergdreef 9, 1105 AZ, Amsterdam, The Netherlands

**Keywords:** Child, Cognition, Pediatric psychology, Cognitive screening, Self report, Parent report

## Abstract

**Objective:**

Efficient screening for neurocognitive dysfunction is pivotal for timely intervention in at-risk populations in pediatrics. The Pediatric Perceived Cognitive Functioning (PedsPCF) item bank was developed for this purpose. We aimed to explore the relationship between, and the discriminative value of PedsPCF scores with neurocognitive outcomes and the behavior rating inventory of executive function parent report (BRIEF) in a pediatric population.

**Methods:**

The PedsPCF parent- and self-report versions were added to neurocognitive testing batteries that were administered in clinical care or research in two Dutch academic pediatric hospitals. Most test batteries were individually tailored, resulting in a wide variety of measures. We determined Pearson correlations between the PedsPCF and neurocognitive test outcomes categorized into five neurocognitive domains as proposed in the Diagnostic and Statistical Manual of Mental Disorders-5, and the BRIEF-parent. Moreover, we assessed the discriminative values of PedsPCF deficit scores (*M* - 1 *SD*) for neurocognitive domain deficits (*M* - 1 *SD*) and the BRIEF-parent.

**Results:**

The PedsPCF was completed by 104 children and 106 parents. The parent-PedsPCF correlated with three neurocognitive domains and the BRIEF-parent but did not indicate deficits in any of the neurocognitive domains. The self-report PedsPCF correlated with a deficit in complex attention and the BRIEF-parent, and could indicate a deficit in complex attention only.

**Conclusions:**

Although the PedsPCF correlated with neurocognitive test outcomes, the discriminative value of the total score was limited. The short and freely available PedsPCF appears to add a useful subjective dimension to neurocognitive testing rather than a replacement of neurocognitive assessment.

## SCREENING FOR NEUROCOGNITIVE DEFICITS IN PEDIATRICS-THE CLINICAL UTILITY OF THE PEDSPCF

Increasing numbers of children grow up with chronic conditions that are associated with neurocognitive deficits (e.g., de Ruiter et al., 2013; Glinianaia et al., 2020). Pediatric conditions such as brain tumors affect neurocognitive functioning (de Ruiter et al., 2013). Moreover, given the developing nature of children’s brains, cognitive challenges may emerge over time. To ensure optimal functioning and development of children with chronic conditions, monitoring cognitive functioning for timely detection and treatment of neurocognitive deficits is essential. However, a comprehensive evaluation with a neurocognitive test battery is time- and cost-intensive. A stepped-care approach, with an initial brief screening for potential neurocognitive problems, and when needed, a more extensive evaluation of cognitive functioning, could be a way to target those children who need a more comprehensive evaluation. Hardy et al. (2017) state that monitoring for neuropsychological problems in at-risk groups should consist of both performance-based tests and “symptom questionnaires”, which should be brief, psychometrically sound, easy to adapt and interpret, and have a clear indication of impairment.

A commonly used neuropsychological functioning (symptom) questionnaire is the Behavior Rating Inventory of Executive Function (BRIEF: Gioia et al., 2000). Although not a screening measure, the BRIEF is a commonly used measure of neuropsychological functioning. However, the BRIEF is considered insufficient to screen the pediatric population, for instance, to screen for cognitive late effects in children with a brain tumor (de Vries et al., 2018). The pediatric perceived cognitive functioning (PedsPCF) item bank was developed to screen for neurocognitive problems in pediatric children (Lai et al., 2011a), and is available for computerized adaptive testing (CAT), for more (time)efficient screening. Items concern real-world cognitive functioning and are scored on problem severity, problem intensity, or both. The PedsPCF item bank is primarily intended for CAT within the Patient-reported outcomes measurement (PROMIS) framework (Lai et al., 2014), where it is known as PROMIS Pediatric Cognitive Function. It has been translated into various languages, including Dutch (Marchal et al., 2019). The PedsPCF shows sound psychometric properties and relates to other symptom questionnaires (Lai et al., 2011b). PedsPCF scores were previously found to discriminate leukoencephalopathy grades (Lai et al., 2017), to relate to both disease symptomatology (Lai et al., 2011b) and neurocognitive outcomes (Lai et al., 2014). Further, the PedsPCF was reported to correlate with processing speed, attention, learning, and working memory of the Cogstate computerized cognitive test battery (Lai et al., 2014). The PedsPCF parent report (but not self-report) was found to relate to neurocognitive outcomes (predominately to intelligence scores) in children with congenital anatomical foregut anomalies and children treated with neonatal extracorporeal membrane oxygenation, more strongly than the BRIEF (Ilik et al., 2022). However, the PedsPCF is less sensitive than the BRIEF in children with portal hypertension (Ohnemus et al., 2019). All in all, the PedsPCF demonstrates robust psychometric properties, and significant associations with various clinical and neurocognitive outcomes have been reported. It is, however, yet to be established whether the PedsPCF could be implemented as an initial screening instrument in clinical practice, i.e., to estimate whether subsequent neuropsychological testing is needed, and whether parent-, self-report, or a combination of the two would be most useful.

With the current study, we aimed to examine the relationship between PedsPCF scores (symptom questionnaire) and neurocognitive outcomes (performance-based tests). First, to study the concurrent validity of the PedsPCF, we tested the correlation of PedsPCF *T-*scores with neurocognitive domain test *T-*scores, and with BRIEF-parent *T-*scores. Second, to study the discriminative value of the PedsPCF, we evaluated whether a *T-*score < 40 (*M* – 1 *SD*) on the PedsPCF was discriminative of deficits in neurocognitive tests (a *T-*score < 40 (M – 1 SD) on one or more neurocognitive domains as defined in the Diagnostic and Statistical Manual of Mental Disorders 5th edition (DSM 5: American Psychiatric Association, 2013)) and the BRIEF parent (a *T-*score > 60; *M* + 1 *SD*). We expected that the PedsPCF would positively correlate with and indicate deficits in neurocognitive tests.

## METHOD

### Participants and procedure

The PedsPCF and sociodemographic questions were added to neuropsychological assessment batteries of two Dutch academic hospitals (Erasmus MC, and two locations of Amsterdam UMC). Three groups of participants were included; 1) Adrenoleukodystrophy (X-ALD) patients who were assessed with a standardized battery for another scientific study, 2) patients who were referred for neuropsychological evaluation by their pediatrician, and 3) children who had been treated for brain tumors and were tested according to the neuropsychological follow-up protocol. Due to this true-to-life approach and the broad age range, there was a great variety of neurocognitive tests. Assessments were performed between June 2015 and June 2018. For an overview of the participants, see Table 2. All parents gave informed consent for the anonymous usage of the clinical data of their child for research purposes. The study was done in accordance with the Helsinki Declaration. Exemption of formal medical ethical evaluation was given by the ethics committee of the AMC (W15_306 # 15.0362), and the ethics committee of the Erasmus MC (MEC-2016-078).

**Table 2:** Characteristics of participants

	**Parent/caregiver**	**Child (*N* = 114)**
**Complete PedsPCF (*n,%*)**	106 (92.9)	104 (91.2)
**Age in years (*M, SD)***	44.8 (5.7)^*a*^	11.8 (3.2)
**Age range in years**	30.3–60.4	7.1–18.1
**Sex assigned at birth**		
Male (*n,%*)	17 (16.0)	66 (57.9)
Female (*n,%*)	68 (64.2)	48 (42.1)
Both parents (*n,%)*	9 (8.5)	
Undisclosed (*n,%*)	1 (0.9)	
Missing (*n,%*)	11 (10.4)	
**Medical diagnosis** ^**b**^		
Oncology (*n,%)*		66 (57.9)
Neurology (*n,%)*		18 (15.8)
Metabolic disorder (*n,%)*		9 (7.9)
Traumatic brain injury (*n,%)*		6 (5.3)
Neonatology (*n,%)*		4 (3.5)
Cardiology (*n,%)*		3 (2.6)
Hematology (*n,%)*		2 (1.8)
Rehabilitation medicine (*n,%)*		2 (1.8)
Immunology (*n,%)*		2 (1.8)
Endocrinology (*n,%)*		1 (0.9)
Audiology (*n,%)*		1 (0.9)
**Psychiatric and Neurodevelopmental Conditions** ^**c**^		
Learning disorder (*n,%)*		10 (8.8)
ADHD (*n,%)*		3 (2.6)
Mood disorder (*n,%)*		2 (1.8)
Autism spectrum disorder (*n,%)*		1 (0.9)
Anxiety disorder (*n,%)*		1 (0.9)

a *n* = 91, for the remaining 15 parents/caregivers the date of birth was missing

b Determined by the disorder as reported by parents/caregivers.

c As reported by parents/caregivers.

### Instruments

Sociodemographic and disease variables were filled in on paper forms by the parents or caregivers (see Table 2).

#### PedsPCF

The PedsPCF (Lai et al., 2011a) is a self-report and parent/caregiver-report item bank screening for neurocognitive problems in pediatric children. It consists of 43 items scored on a 5-point Likert Scale; 13 of these items overlap in phrasing but differ in answering scale. The PedsPCF has been translated to Dutch and validated in the Netherlands (Marchal et al., 2019). In the current analyses, only the 30 unique items were used, in line with Dutch norm data (Marchal et al., 2019). The PedsPCF measures functioning rather than dysfunction and results in a total score given its unidimensional character with a higher score indicating better functioning.

#### BRIEF

The BRIEF-parent questionnaire measures executive functioning (Gioia et al., 2000; Huizinga & Smidts, 2009). It includes 65 items, resulting in a Total score and two index scores (Behavioral Regulation and Metacognition), with higher scores indicating worse functioning. It shows high internal consistency and sufficient test–retest reliability (Gioia et al., 2000). The BRIEF-parent was not completed for every child, but only if it was part of the protocolized test battery, or when selected by the psychologist in a tailored battery.

#### Neurocognitive tests

For each patient, a battery was composed based on the clinical relevance (e.g., referral, age, condition). This individual approach resulted in a wide variety of employed materials. Descriptions of the measures that were used can be found in Table 1. The measures were categorized by neurocognitive domain as defined in the DSM-5 (American Psychiatric Association, 2013): Attention, Executive Functioning, Perceptual-Motor functioning, Language, and Learning and Memory. Measures were categorized by JPM, MDV, and KO individually based on the measurement objectives. Consensus was reached where categorization differed between evaluators. Descriptions of the used measures can be found in Table 1.

**Table 1:** Overview of all used neurocognitive tests

Cognitive function	Measure	Included score and description
Complex Attention	D2 Test of Attention (Brickenkamp & Zillmer, 1998)	**Concentration performance score** Identify targets (a “d” with two dots) among distractors (e.g., “d” and “p” with dots) (pencil and paper).
	Stroop Colour-Word test (Hammes, 1978)	**Color** Name the colors on a sheet with colors (blue, red, yellow). **Word** Read the word on a sheet with color names (blue, red, yellow) written in black.
	Test of Everyday Attention for Children (TEA-Ch) (Manly et al., 1999))	**Sky search** Find target spaceships among distractor spaceships**.** **Score!** Count target tones at varying intervals from an audiotape. **Creature counting** Count forward and backward in response to visual instructions on target stimuli. **Sky Search Double Task (DT)** Do Sky Search and Score! simultaneously. **Map mission** Find targets among distractors on a city map. **Score! DT** Perform Score! and simultaneously perform distill an animal name from an auditory text. **Walk, Don’t Walk** Follow steps on a paper, but inhibit this when a tone is heard on an audio tape. **Opposite worlds** On a list with 1 s and 2 s, state 1 when 2 is written and vice versa. **Code transmission** Distill target numbers from a list of auditory numbers.
	Trail Making Test (Bowie & Harvey, 2006)	**Part A** Connect the dots of ascending numbers, 1–2–3-4.
	Vienna Reaction Test (Schuhfried, 1992)	**Test S1** Press a specified key in response to a specified stimulus (visual).
	Wechsler Intelligence Scale for Children-III (WISC-III) or Wechsler Adult Intelligence Scale-III (WAIS-III) Processing speed subtests (Wechsler, 1997; Wechsler, 2003)	**Coding** Specified symbols have to be written under a series of numbers. **Symbol search** A specified symbol has to be searched among a range of symbols.
Executive Function	Behavioural Assessment of the Dysexecutive Syndrome for Children (BADS-C) (Wilson et al., 2003)	**Playing Cards Test** In response to demonstrated cards 1) respond “yes” to red, and “no” to black cards, and 2) respond “yes” to two consecutive cards with the same color, and “no” to dissimilar colored cards (*Cognitive Flexibility*). **Water Test** Retrieve a cork from a tube with several available items (*Problem Solving*). **Key Search Test** Draw on a white square how to search for a lost key in a field (*Planning*). **Zoo Map Test 1** 1) On a Zoo map plan a route to see specified animals. 2) Follow a written plan on the same Zoo map (*Planning*). **Six Part Test** Within 5 minutes children have to do 2 sorting, 2 arithmetic, and 2 picture naming tasks, not doing two similar tasks in sequence (*Planning, performance monitoring*).
	Stroop Colour-Word test (Hammes, 1978)	**Interference** Name the color that a word is printed in on a sheet with color names (blue, red, yellow) written in colored ink, without reading the word.
	Trail Making Test (Bowie & Harvey, 2006)	**Part B** Connect the dots of, alternating, the order of ascending numbers and the alphabet, i.e., 1-A-2-B-3-C. **Part B-A** time taken on TMT-B minus time taken on TMT-A.
	Vienna Reaction Test (Schuhfried, 1992)	**Test S3** A computerized test/assessment battery. The Reaction Tests measure attention through reaction time to visual and auditory stimuli, which is measured by pressing a specified key (choice) in response to a specified stimulus (visual and auditory). Tests 13 were administered. On test 1, react to a yellow light, on test 2, react to a tone, on test 3 react to the light and tone together.
Perceptual-motor Function	Beery Visual Motor Integration, Fifth edition (Beery, 1997)	**Visual Motor Integration** Several pictures have to be copied (pencil and paper).
	Rey Complex Figure Test (Meyers & Meyers, 1995)	**Direct Copy** A complex figure has to be copied (pencil and paper).
	Selected WISC-III or WAIS-III perceptual organization subtests	**Picture Completion** On a picture one has to point out what is missing. **Block Design** A 2-D picture has to be copied with red and white colored blocks. **Object Assembly** A jigsaw puzzle has to be completed (WISC-III only) **Maze** Several paper and pencil mazes have to be completed (WISC-III only)
Language	WISC-III or WAIS-III Verbal Comprehension subtests	**Information** Factual questions. **Similarities** The similarity between two cases has to be described. **Vocabulary** Words have to be explained. **Comprehension** Questions about social constructs and everyday concepts.
Learning & Memory	NEPSY-II Narrative Memory (Korkman et al., 2007)	**Free and Cued Recall score** After listening to a story, repeat the story, and answer questions on missing details from recall.
	Rey Complex Figure Test (Meyers & Meyers, 1995)	**Immediate & Delayed Recall** Draw the complex figure from memory, immediately after copying the figure and again after 30 minutes.
	15 Words Test (Rey, 1964)	**Immediate & Delayed recall** A list of 15 words is read out loud, and these words have to be repeated from memory immediately and after 15–20 minutes.
	WISC-III or WAIS-III subtests	**Digit Span** An increasing sequence of numbers had to be verbally repeated forward and backwards. **Letter Number sequencing** An increasing sequence of letters has to be repeated in the correct order (small to large/alphabetically) (WAIS-III only)
	Wechsler Memory Scale (Wechsler, 2009)	**Logical Memory (I, II)** recall details from short stories, both immediately and after a delay. **Verbal Paired Associates (I,II)** recall of word pairs after hearing them once or multiple times **Designs (I, II)** reproduce abstract designs after a brief exposure **Visual Reproduction (I, II)** recall geometric figures both immediately and after a delay **Symbol Span** recall sequences of abstract symbols in the correct order **Spatial Addition** mentally manipulate and combine visual–spatial information

### Statistical analyses

Missing values of the PedsPCF were imputed by the mean score on the completed items, with a maximum of 6 items missing. If more items were missing, the participant was excluded from the analyses. If multiple answers were given on one item, the most severe answer option was selected. Internal consistency was assessed by Guttman’s Lambda 2 where ≥0.80 was deemed good, and ≥ 0.90 as excellent (Cohen, 1988). PedsPCF sum scores were transformed into *T-*scores based on the mean and *SD* of the norm sample based on a large general population sample (Marchal et al., 2019).

For each neurocognitive domain the scores of (sub)tests, as listed in Table 1, were transformed to *z*-scores and subsequently to *T-*scores based on the current data, after which a mean *T-*score was calculated for all administered subtests within the domain. For all measures, a score of 1 *SD* above or below the mean (*T-*score of 40 or 60, dependent on whether a higher or lower score reflected poorer functioning) was used as an estimate of deficits in neurocognitive functioning. For descriptive purposes, the *T-*scores of the PedsPCF, the neurocognitive domain *T-*scores, and BRIEF-parent *T-*scores were compared with norm data (*M* 50, *SD* 10) using one-sample *T-*tests, two-tailed.

First, to assess concurrent validity, we determined Pearson’s correlations between PedsPCF *T-*scores, the neurocognitive domain scores, and BRIEF-parent scores, respectively, considering an *r* of 0.10 to be small, 0.30 medium, and 0.50 large (Cohen, 1988).

Second, to determine the extent to which PedsPCF deficit scores (*T-*score < 40) indicated deficits in neurocognitive domains (*T-*score < 40), several Chi-square analyses were done: 1) for the PedsPCF parent- and self-report separately, 2) for combinations of PedsPCF deficit scores (a *T-*score < 40 on either the parent or the self-report, or a *T-*score < 40 on both). 3) whether PedsPCF scores indicated a deficit (i.e., a *T-*score < 40) in *any* neurocognitive domain, 4) whether PedsPCF deficit scores could indicate *the number* of neurocognitive domains showing a deficit score (i.e., the number of domains with a *T-*score < 40), and 5) whether PedsPCF deficit scores indicated BRIEF-parent deficit scores (*T-*score > 60^1^). To get an impression of the discriminative value of the PedsPCF, Chi-square tests were conducted.

## RESULTS

### Participants

A total of 114 children were included, with 106 parents/caregivers completing the PedsPCF and 104 children completing the PedsPCF (See Table 2). For 96 children, both the parent- and self-report PedsPCF were completed. More mothers than fathers completed the PedsPCF. A wide variety of diagnoses were included, yet the majority of children were diagnosed with an oncological disorder (57.9%). For a full overview of socio-demographics and disease characteristics, see Table 2.

### Descriptive statistics

Internal consistency was excellent for both the PedsPCF parent-report and the self-report, with Guttman’s Lambda’s of 0.96 and 0.95, respectively. Both the self-report (medium effect size) and parent-report (large effect size) PedsPCF scores of the current sample were significantly lower than those in the norm sample (see Table 3). Apart from the Language domains, in all neurocognitive domains, scores were lower than the norm. Even though the score on the BRIEF-parent did not differ from the norm scores, it is close to showing a significantly better score in our sample (i.e., a lower *T-*score) compared with the norm.

**Table 3:** PedsPCF, neurocognitive test outcomes, and BRIEF total scores compared with norm data (M 50, SD 10) by one-sample T-tests, two-tailed

	***n***	***T*-score (*M*, *SD*)**	***p***	**Effect size *d***
**PedsPCF**				
Parent-report	106	40.6 (11.9)	<0.001	0.79
Self-report	104	44.8 (11.6)	<0.001	0.45
**Neurocognitive assessments**				
Complex attention domain	110	45.2 (7.9)	<0.001	0.60
Executive functioning domain	77	44.4 (9.0)	<0.001	0.62
Perceptual motor domain	111	44.7 (7.5)	<0.001	0.70
Language domain	106	50.4 (8.4)	0.59	0.05
Learning & memory domain	105	46.2 (9.2)	<0.001	0.41
**Questionnaires**				
BRIEF parent report	70	47.4 (11.6)	0.06	0.23

### Concurrent validity of the PedsPCF

The parent-report PedsPCF showed significant weak correlations with three neurocognitive domain scores (Complex attention, Language, Learning & Memory) and a strong significant correlation with the BRIEF-parent score, all in the expected directions (See Table 4). The self-report PedsPCF showed a weak correlation with the Complex Attention domain and a moderate correlation with the BRIEF-parent scores, again in the expected directions.^2^

**Table 4:** Pearson correlations of PedsPCF with neurocognitive test outcomes and BRIEF total scores

	**Parent-report**		**Self-report**	
	***n***	***r***	***n***	***r***
**Neurocognitive assessments**				
Complex attention domain	102	0.23^*^	100	0.27^**^
Executive functioning domain	71	0.09	72	0.21
Perceptual motor domain	103	0.02	101	0.10
Language domain	98	0.21^*^	97	0.14
Learning & memory domain	97	0.24^*^	96	0.17
**Questionnaires**				
BRIEF parent report total score^a^	66	−0.64^***^	65	−0.44^***^

^*^ *P* < .05, ^**^  *P* < .01, ^***^  *P* < .001

a A higher score on the BRIEF indicates more executive function problems, i.e., an expected lower score on the PedsPCF which concerns overall cognitive functioning

### Discriminative value of the PedsPCF

A deficit score on the parent-report PedsPCF was not significantly discriminative of neurocognitive test deficits (*P* = .14 to .81), whereas it did indicate a deficit score on the BRIEF-parent (*P* = .03) (Table 5). A deficit score on the self-report PedsPCF only indicated a deficit in Complex attention (*P* = .03), but in none of the other domains (*P* = .14 to .96), nor a deficit score on the BRIEF-parent (*P* = .05). A score below the cut-off on *either* the parent- or self-report PedsPCF indicated only deficit scores in Complex attention (*P* = .03) and the BRIEF-parent (*P* = .03). A score below the cut-off on *both* the parent- and self-report PedsPCF only indicated deficit scores for the BRIEF-parent (*P* = .03), and not for any of the neurocognitive domains (*P* = .11 to .80).^3^

**Table 5:** Discriminative value of the PedsPCF

	PedsPCF Parent report				PedsPCF Self-report				PedsPCF				PedsPCF			
	Normal (*n*,%)	Deficit (*n*,%)	*p*	*Χ* ^2^, *df*	Normal (*n*,%)	Deficit (*n*,%)	*p*	*Χ* ^2^, *df*	Parent and self-report normal (*n*,%)	Parent and/or self-report deficit (*n*,%)	*p*	*Χ* ^2^, *df*	Parent and/or self-report normal (*n*,%)	Parent and self-report deficit (*n*,%)	*p*	*Χ* ^2^, *df*
Combined domains			0.81	0.1, 1			0.14	2.2, 1			0.09	2.9, 1			0.80	0.1, 1
All normal	22 (21.2)	20 (19.2)			32 (31.4)	12 (11.8)			21 (20.0)	21 (20.0)			33 (32.7)	11 (10.9)		
One or more deficit	31 (29.8)	31 (29.8)			34 (33.3)	24 (23.5)			21 (20.0)	42 (40.0)			44 (43.6)	13 (12.9)		
Combined domains			0.34	4.6, 4			0.18	6.3, 4			0.11	7.5, 4			0.56	3.0, 4
All normal	22 (21.2)	20 (19.2)			32 (31.4)	12 (11.8)			21 (20)	21 (20)			33 (32.7)	11 (10.9)		
One deficit	15 (14.4)	14 (13.5)			16 (15.7)	11 (10.8)			12 (11.4)	17 (16.2)			19 (18.8)	8 (7.9)		
Two deficits	11 (10.6)	9 (8.7)			13 (12.7)	6 (5.9)			8 (7.6)	13 (12.4)			16 (15.8)	2 (2.0)		
Three deficits	3 (2.9)	8 (7.7)			5 (4.9)	5 (4.9)			1 (1.0)	10 (9.5)			7 (6.9)	3 (3.0)		
Four deficits	2 (1.9)	0 (0)			0 (0)	2 (2.0)			0 (0)	2 (1.9)			2 (2.0)	0 (0)		
Complex attention			0.14	2.2, 1			0.03^*^^a^	4.9, 1			0.03^*^^b^	4.7, 1			0.11	2.5, 1
Normal	42 (41.2)	34 (33.3)			52 (52.0)	22 (22.0)			35 (34.0)	41 (39.8)			59 (59.6)	15 (15.2)		
Deficit	10 (9.8)	16 (15.7)			12 (12.0)	14 (14.0)			6 (5.8)	21 (20.4)			16 (16.2)	9 (9.1)		
Executive functioning			0.72	0.1, 1			0.30	1.1, 1			0.66	0.2, 1			0.80	0.1, 1
Normal	24 (33.8)	21 (29.6)			32 (44.4)	16 (22.2)			21 (28.4)	27 (36.5)			35 (50.7)	10 (14.5)		
Deficit	15 (21.1)	11 (15.5)			13 (18.1)	11 (15.3)			10 (13.5)	16 (21.6)			18 (26.1)	6 (8.7)		
Perceptual motor			0.53	0.4, 1			0.56	0.3, 1			0.24	1.4, 1			0.16	2.0, 1
Normal	38 (36.9)	40 (38.8)			52 (51.5)	27 (26.7)			34 (32.7)	46 (44.2)			56 (56.0)	21 (21.0)		
Deficit	14 (13.6)	11 (10.7)			13 (12.9)	9 (8.9)			7 (6.7)	17 (16.3)			20 (20.0)	3 (3.0)		
Language			0.46	0.5, 1			0.96	0.0, 1			0.17	1.9, 1			0.40	0.7, 1
Normal	46 (46.9)	42 (42.9)			58 (59.8)	30 (30.9)			38 (38.0)	52 (52.0)			66 (69.5)	20 (21.1)		
Deficit	4 (4.1)	6 (6.1)			6 (6.2)	3 (3.1)			2 (2.0)	8 (8.0)			8 (8.4)	1 (1.1)		
Learning & memory			0.77	0.1, 1			0.46	0.5, 1			0.07	3.3, 1			0.14	2.2, 1
Normal	38 (39.2)	36 (37.1)			50 (52.1)	24 (25.0)			33 (33.3)	41 (41.4)			55 (58.5)	19 (20.2)		
Deficit	11 (11.3)	12 (12.4)			13 (13.5)	9 (9.4)			6 (6.1)	19 (19.2)			18 (19.1)	2 (2.1)		
BRIEF			0.03^*^	4.6, 1			0.05	3.8, 1			0.03^*^	4.9, 1			0.03^*^	4.5, 1
Normal	35 (52.2)	21 (31.3)			42 (63.6)	12 (18.2)			29 (42.6)	27 (39.7)			48 (73.8)	6 (9.2)		
Deficit	3 (4.5)	8 (11.9)			6 (9.1)	6 (9.1)			2 (2.9)	10 (14.7)			7 (10.8)	4 (6.2)		

*Discriminative value of the PedsPCF*

^*^ *P* < .05

a For complex attention deficits, the PedsPCF Self-report showed a sensitivity of 53.9% [33.4–73.4%], a specificity of 70.3% [58.5–80.3%], a positive predictive value of 38.9% [27.9–51.2%] and a negative predictive value of 81.3% [73.6–87.1%]

b For complex attention deficits, this combined score of the PedsPCF Parent-report and Self-report showed a sensitivity of 77.8% [57.7–91.4%], a specificity of 46.1% [34.6–57.9%], a positive predictive value of 33.9% [27.2–40.6%] and a negative predictive value of 85.4% [73.4–92.5%]

*Note*. Percentages are based on the total cases in each analysis. A deficit score on the PedsPCF is defined as a *T*-score < 40, a deficit score on cognitive domains is defined as a mean *T*-score < 40, and a deficit score on the BRIEF parent is defined as a T-score > 60.

## DISCUSSION

This study describes the relationship between the PedsPCF, neurocognitive tests, and the BRIEF-parent. Doing this in a naturalistic setting, we aimed to explore the potential role of the PedsPCF in screening for cognitive difficulties in clinical practice. The results indicated limited discriminative value of the PedsPCF for neurocognitive test outcomes. Although the PedsPCF correlated with some neurocognitive test outcomes, it hardly indicated deficit scores on neurocognitive domains. The PedsPCF did correlate with and indicated deficit scores for the BRIEF-parent, an established neurocognitive symptoms questionnaire, however, this would not survive Bonferroni correction.

Our findings extend previous findings on the relationship between the PedsPCF-parent and neurocognitive testing outcomes. In line with Lai et al. (2014) and Ilik et al. (2022), who found that the PedsPCF-parent was associated with neuropsychological test outcomes, we found that the PedsPCF-parent correlated with certain neurocognitive outcomes (complex attention, language, and learning & memory). However, despite these promising initial findings, the PedsPCF-parent did not *indicate* dysfunction in any of these domains (i.e., the PedsPCF deficit scores did not align with deficit scores on the neurocognitive domains).

Our study extends previous studies by exploring parent and self-report PedsPCF. Lai et al. (2014) did not analyze self-reports of the PedsPCF, and Ilik et al. (2022) found no significant relationships between the self-report version and neurocognitive testing outcomes. Our results show only a relationship and discriminative value of the PedsPCF-self with complex attention. The clinical utility of this discriminative value is minimal, as a deficit score on the PedsPCF only identified 15 of the 27 children with a deficit score on the complex attention domain. Further, 22 of the 37 children with a clinical score on the PedsPCF-self had a normal complex attention score.

A new angle in this study is to assess the combination of self- and parent-report on the PedsPCF. From both variations we studied, it appeared more productive to establish whether either the parent or the child reported a clinical score on the PedsPCF, than both the parent and the child. However, the combination of the parent- and self-report PedsPCF (either self *or* parent scoring below the cut-off) could still only indicate deficits in complex attention. Most children with a deficit score in this domain (22 of 28 children) are correctly identified using the PedsPCF parent- and self-report. This suggests that using the PedsPCF-self in combination with the PedsPCF-parent could potentially be discriminative for a single neurocognitive subdomain, but cannot be used as a stand-alone measure to screen for broader neurocognitive difficulties. This aligns with previous research showing that parent-reported EF and cognitive testing often correlate poorly (de Vries et al., 2018), and seem to measure different constructs (Ten Eycke & Dewey, 2016).

For clinical use, our findings imply that while PedsPCF scores may correlate with neurocognitive outcomes, their discriminative value for such outcomes is limited. As stated in the introduction, Hardy et al. (2017) described the complementary use of performance-based tests and symptom questionnaires in screening for neuropsychological problems in at-risk groups. Although questionnaires might be influenced by interpretation and understanding of the questions, the current state of mind, stress, psychiatric condition, and reading capability (Roebuck-Spencer et al., 2017), they do give important information about subjective neurocognitive difficulties in daily life, which relate to quality of life (de Vries & Geurts, 2015). For example, a child who has regressed neurocognitive functioning would report neurocognitive difficulties, even though they might still score within the “normal” range when tested with neurocognitive tests. In short, although our findings suggest that the PedsPCF cannot be used as the sole measure in a stepped-care approach in a broad and diverse pediatric population (i.e., to evaluate who should or should not undergo extensive neuropsychological examination), they do suggest that the PedsPCF adds an important subjective dimension to the neurocognitive testing outcomes.

The substantial relationship between the PedsPCF and the BRIEF-parent does imply that the PedsPCF measures a similar construct. The current findings suggest that the PedsPCF is more sensitive to cognitive deficits in this particular section of pediatrics since the BRIEF-parent scores did not differ from the norm data, and the PedsPCF did, in line with Ilik et al., (2022). This aligns with the intended purpose of the questionnaires; the PedsPCF was developed to measure broader cognitive functioning in the pediatric population, while the BRIEF-parent was developed to measure executive functioning. Possibly, there was a response shift (i.e., a change in self-evaluation resulting from a change in the respondent’s standards regarding the measured construct) on the BRIEF-parent, which does not happen on the PedsPCF. The choice for one or the other measure depends on the aim of the assessment. The PedsPCF does have the advantage that it can be used as a CAT measure. CAT measures allow for brief, tailored assessment by minimizing the number of questions for each individual, resulting in a clear score on a single dimension. Moreover, the PedsPCF is available for healthcare in the Netherlands as PROMIS Pediatric Cognitive Function, making it easy to use in screening protocols.

### Limitations

Our study does have its limitations. Firstly, the neurocognitive test data were heterogeneous due to data collection in clinical practice. Some domain scores were, therefore, reflected by different tests (e.g., TEA-Ch vs D2). Some tests were relatively old, given the use of validated instruments in Dutch, which are published with a lag compared to the original publications. However, these heterogeneous data reflect neuropsychological testing in the clinical setting, making the current results more ecologically valid compared to standardized scientific test batteries (see Lai et al., 2014). Secondly, there was a possible effect of selection bias. While our primary interest concerns the utility of the PedsPCF in screening pediatric groups of children at risk for neurocognitive problems, the majority of children in the current study were *referred* for comprehensive neuropsychological testing. This might partly explain the limited discriminative value, as most children in the current sample were expected to have neurocognitive difficulties. Moreover, whether our findings apply to children at risk, but not referred for full evaluation, remains unclear. Also, our findings are based on children with sufficient language abilities to fill in the PedsPCF. This may be one of the reasons we found relatively few children with deficit scores on the language tests. This limited the power of our analysis of the predictive power of the PedsPCF for language deficits and likely also limits the extent to which our findings can be generalized to children with poorer language abilities. We did not collect data on language ability, hence, we could not evaluate its possible influence. Thirdly, for the PedsPCF we used *T-*scores based on Dutch norm data, for the neurocognitive domains, we used a cut-off of 1 *SD* from the population mean. This is a somewhat arbitrary way to define cognitive dysfunction. For this study, this was the most consistent way of analyzing all data (given the variety of measures used for the neurocognitive domains), but for clinical practice, we advise adherence to cut-offs from the manuals or literature for each instrument. For the PedsPCF, further evaluation of the currently used cut-off of -1*SD* has previously been recommended (Lai et al., 2017). Based on our current findings, it is debatable whether this is the best approach to predict neurocognitive outcomes. Another limitation is that we evaluated the PedsPCF as a unidimensional measure, with a single neurocognitive score, while the tests were all subdivided into subdomains. We chose this approach since the PedsPCF is used as PROMIS Pediatric Cognitive Function, which assumes one-dimensionality (Marchal et al., 2019). However, we would argue that condensing neurocognitive functioning into one domain does not do justice to the multidimensional construct that is split into six domains in the DSM-5, an authoritative guide for clinical practice. Finally, our test batteries did not include performance validity tests; while these are recommended in the literature, they are not frequently used in our clinical setting (Holcomb, 2018).

### Future directions

Future studies could look into the discriminative value of the PedsPCF in more structured neurocognitive test batteries. Moreover, multiple testing to evaluate test–retest reliability and the possible usefulness of the PedsPCF to monitor the progression of experienced neurocognitive symptoms or cognitive development over time (as suggested in Roebuck-Spencer et al., 2017) may be evaluated. Second, teachers are a valuable source of information concerning neurocognitive functioning (de Vries et al., 2018), suggesting that the PedsPCF-teacher might be a useful additional tool to measure neurocognitive functioning at school. Third, it might be fruitful to study whether using subscales reflecting neurocognitive subdomains would be a better approach to using the PedsPCF, or whether the PedsPCF could provide important information for intakes. Moreover, using the categorization of neurocognitive domains according to the DSM-5 (APA, 2013) was a well-considered but debatable and maybe even arbitrary choice. Alternative categorizations are possible, and many tasks tap into several cognitive domains. For example, tasks that measure Complex Attention also reflect processing speed. Possibly specific subscales relate to specific cognitive domains, but this is challenging to map accordingly. Furthermore, there are indications that the PedsPCF does relate to intellectual and academic difficulties (Wolfe et al., 2022), suggesting that the PedsPCF might relate better to other domains than those currently studied. Fourth, adding validity indices to or alongside the PedsPCF might be a useful addition to the PedsPCF. Finally, it would be important to study whether the PedsPCF would relate better to daily life support needs than to the current tasks and tests.

To conclude, our study provides important knowledge about using the PedsPCF in clinical practice. As a CAT measure, it might be a valuable, brief, and easily employable instrument to assess this subjective dimension of neurocognitive problems in screening protocols for at-risk pediatric populations. Combining parent and self-reports might be more informative than single informant information. However, our findings indicate that the PedsPCF cannot replace neurocognitive testing nor predict who would benefit from it, but might provide additional information about perceived (subjective) neurocognitive challenges.

## Footnotes

1 Note that a higher BRIEF-score indicates more EF problems. Note that the cut-off score we used in the present study differs from the cut-off score in the BRIEF manual (*T-*score > 65), to conform with the used cut-off scores for all other measures.

2 Given the explorative character of the analyses, we did not apply Bonferroni Correction. However, had we applied Bonferroni correction for multiple comparisons (α = 0.05/6 = 0.008), only the relationship between the Peds-PCF self-report and the Complex Attention Domain and the PedsPCF parent and self-reports with the BRIEF would have been significant.

3 None of the discriminatives would be significant after Bonferroni corrections.
